# Dietary supplementation with the extract from *Eucommia ulmoides* leaves changed epithelial restitution and gut microbial community and composition of weanling piglets

**DOI:** 10.1371/journal.pone.0223002

**Published:** 2019-09-26

**Authors:** Mijun Peng, Zhihong Wang, Sheng Peng, Minglong Zhang, Yehui Duan, Fengna Li, Shuyun Shi, Qiuling Yang, Changwei Zhang

**Affiliations:** 1 Guangdong Provincial Public Laboratory of Analysis and Testing Technology, Guangdong Institute of Analysis, Guangzhou, P. R. China; 2 National & Local United Engineering Laboratory of Integrative Utilization Technology of *Eucommia ulmoides*, Jishou University, Zhangjiajie, P. R. China; 3 Key Laboratory of Agro-ecological Processes in Subtropical Region, Institute of Subtropical Agriculture, Chinese Academy of Sciences, National Engineering Laboratory for Pollution Control and Waste Utilization in Livestock and Poultry Production, Hunan Provincial Engineering Research Center for Healthy Livestock and Poultry Production, Scientific Observing and Experimental Station of Animal Nutrition and Feed Science in South-Central, Ministry of Agriculture, Changsha, P. R. China; 4 Hunan Co-Innovation Center of Animal Production Safety, CICAPS, Hunan Collaborative Innovation Center for Utilization of Botanical Functional Ingredients, Changsha, Hunan, China; 5 College of Chemistry and Chemical Engineer, Central South University, Changsha, Hunan, China; 6 Institute of Chemical Industry of Forest Products, CAF, Nanjing, P. R. China; University of Illinois, UNITED STATES

## Abstract

This study was conducted to compare the effects of *Eucommia ulmoides* leaves (EL) in different forms (EL extract, fermented EL, and EL powder) with antibiotics on growth performance, intestinal morphology, and the microbiota composition and diversity of weanling piglets. Compared to the control group, the antibiotics and EL extract significantly increased the average daily gain and decreased the feed: gain ratio as well as the diarrhea rate (*P* < 0.05). The EL extract significantly decreased the crypt depth and increased the ratio of villus height to crypt depth (*P* < 0.05), while the fermented EL group did the opposite (*P* < 0.05). The crypt depth in the antibiotics group was of similar value to the EL extract group, and was lower than the fermented EL and EL powder groups (*P* < 0.05). Compared to the control and antibiotics groups, the jejunul claudin-3 mRNA expression and the concentrations of total VFA, Chao 1, and ACE were significantly augmented in the EL extract group of piglets (*P* < 0.05). The EL extract groups also showed elevated Shannon (*P* < 0.05) and Simpson (*P* = 0.07) values relative to the control and antibiotics groups. At the phylum level, the EL extract group exhibited a reduced abundance of *Bacteroidetes* and an enhanced abundance of *Firmicutes*. At the genus level, the abundance of *Prevotella* was augmented in the EL extract group. Moreover, compared with the antibiotic group, the acetate concentration was enhanced in the EL extract and fermented EL groups. Overall, dietary supplementation with the EL extract, but not the fermented EL or EL powder, improved growth performance, jejunul morphology and function, as well as changed colonic microbial composition and diversity, which might be an alternative to confer protection against weanling stress in weanling piglets.

## Introduction

Antibiotics as feed additives is well recognized to confer protection against diseases and to improve growth performance in swine production [1, 2]. Despite these positive outcomes, an increasing body of literature reports that antibiotics are increasingly abused, which may greatly promoted the development of drug-resistant bacteria [3]. What's more frightening is that these drug-resistant bacteria and their resistant factors may be transferred from animals to humans [4]. Based on the above disadvantages, the use of antibiotics as feed additives in swine production has been banned by the European Union, the United States, and more and more countries. However, with the implementation of the ban of in-feed use of antibiotics, the high incidence of diseases occurred [4]. This will exert avoidable consequences for growth performance of animal production. Therefore, to overcome the increased rate of mortality and morbidity, an intensive search for replacements/alternatives has become a hot area of research in the last decade.

Among potential candidates, herbal plants, which are used widely in traditional systems of medicine, have recently gained popularity as a new class of growth promoters [5]. Compared with inorganic chemicals or synthetic antibiotics, these plant-derived products have proven to be natural, safe and less toxic, residue free, and effective against certain bacteria [6], and are viewed as ideal growth promoters in animal diets, particularly in Asian, African, and South American countries [2, 7]. *Eucommia ulmoides* leaves (EL) are rather ubiquitous as the by-product of traditional Chinese medicinal herb Du-zhong. EL is rich in bioactive compounds (such as flavonoids, chlorogenic acid, aucubin, and geniposidic acid) and has anti-inflammatory, antioxidant, antiviral and hepatoprotective functions [8–12]. Therefore, EL is regarded to be a beneficial antioxidant feed additive. Despite these positive outcomes, very few studies concerning the comparisons of EL with antibiotics on the animal gut microbiota have so far been conducted. Moreover, it is still uncertain that which form of EL (EL extract, fermented EL, or EL powder) is most effective.

We hypothesized that the different form of EL exerted different roles in regulating growth performance and intestinal health in weaned pigs. Therefore, this study aimed to investigate the effects of dietary supplementation of different forms of EL on the growth performance, intestinal morphology, tight junction protein expression, as well as the colonic microbiota composition and diversity of weaned pigs, and anticipate to provide a reference for the application of EL in weaned piglets without antibiotic-feed.

## Materials and methods

### Materials

*Eucommia ulmoides* leaves (EL) were collected from Cili Du-zhong forestry centre (Zhangjiajie, China), and was air-dried, processed, and analyzed as previously described [13]. The content of total flavonoids, chlorogenic acid, aucubin, and geniposidic acid in EL powder is 7.01%, 2.13%, 4.42%, and 5.39%, respectively. In addition, its extract (polysaccharides > 20.00%, total flavonoids > 8.00%, chlorogenic acid > 5.00%) was obtained through spray drying for aqueous extract of EL. The fermented EL was prepared as previously described [14]. Its effective constituents are as follows: water (5.80%), geniposidic acid (1.99%), total flavonoids (4.30%), chlorogenic acid (1.23%), and streptococcus lactate (3.6 × 10^8^ CFU/g).

### Determination of active ingredients

Total flavonoid content was determined using a previous colorimetric method with slight modification and calculated using a rutin as standard [15]. A total of 0.5mL of sample solution was placed in a 10mL volumetric flask. Then 0.3mL of 5% NaNO_2_ solution was added, and 0.3 mL of 10% Al(NO_3_)_3_ solution was added later 6.0 min, and the mixed solution was allowed to stand for 6 minutes. Subsequently, 4.0 ml of a 1.0 mol/L NaOH solution was mixed with the above solution, and the reaction solution was made up to the mark with distilled water. It was thoroughly mixed and placed for 15min before testing. The absorbance was measured at 506nm with the spectrophotometer.

The content of chlorogenic acid, geniposidic acid and aucubin were estimated according to the previous report [16]. Analyses of the standard and sample solution were carried out using LC-20A HPLC instrument (SHIMADZU, Japan) with Thermo BDS HYPERSIL C_18_ column (250 mm×4. 6 mm, 5 μm).The mobile phase consisted of (A) water containing 0.1% formic acid and (B) methanol. The gradient elution had the following profile: 0–5 min, 15% B; 5–18 min, 15–30% B; 18–23 min, 30% B; 23–24 min, 30–35% B; 24–29 min, 35–15% B; 29–30 min, 15% B. The flow rate was 1.0 mL/min. The injection volume was 10 μL. UV spectra recorded was in the range of 195–400 nm.

The content of the polysaccharides was measured by phenol sulfuric acid methods with slight modification [17]. 1.0mL sample solution was added to the test tube with plug, 1.0mL 5.0% phenol solution and sample solution were mixed in the test tube, then 5.0mL concentrated sulfuric acid was quickly added. The mixed reaction solution was incubated at 40°C water bath for 30 min at constant temperature. The system was cooled for 15 min and its absorbance was measured at 490 nm. Distilled water was used as a blank group, and a calibration curve was obtained using glucose as a standard.

### Animals and diets

All animal procedures were performed in strict accordance with the recommendations of the Guide for the Care and Use of Laboratory Animals of the Guangdong Institute of Analysis, and was approved by the Committee on the Ethics of Animal Experiments of the Guangdong Institute of Analysis under ethic approval number SYXK 2019–0201.

A total of fifty weaned piglets (Landrace × Large white × Duroc, 21 ± 2 d, barrow) with a similar initial body weight (7.22 ± 0.34 kg) were randomly allocated into five groups with 10 replicates per group and one piglet per replicate in a completely randomized design according to the body weight. Piglets in each group received either a basal diet with no supplement (control), or a diet supplemented with antibiotic (75 mg/kg chlortetracycline), 0.50% EL extract, 6.00% fermented EL, or 6.00% EL powder. All diets were designed to fulfill nutrient requirements according to the National Research Council [18] (as shown in **Table 1**). The dietary crude protein, calcium (Ca), and phosphorus (P) were measured according to previous methods [19]. The amino acid compositions in diets were analyzed as previously described [20]. All pigs were individually housed in cages (1.0 × 0.8 m) with a single-hole feeder and 1 nipple drinker. The pigs had *ad libitum* access to diets and water and consumed the diets for 28 days.

**Table 1:** Composition and nutrient levels of the basic diets (air-dried basis).

Ingredients	Content (g/kg)
Corn	637
Soybean meal	198
Dried whey	43
Fish meal	90
Soybean oil	8
Lys	3.8
Met	1
Thr	0.9
Trp	0.1
CaHPO_4_	0.0
Limestone	5.2
Salt	3
Premix^1^	10
Nutrient content (%)	
DE (MJ/kg)^2^	14.61
CP	200.5
Lys	12.3
Met + Cys	6.8
Thr	7.3
Trp	1.9
Total Ca	7
Total P	5.7

^1)^Supplied per kg of diet: vitamin A, 10 800 IU; vitamin D3, 4000 IU; vitamin E, 40 IU; vitamin K3, 4 mg; vitamin B1, 6 mg; vitamin B2, 12 mg; vitamin B6, 6 mg; vitamin B12, 0.05 mg; biotin, 0.2 mg; folic acid, 2 mg; niacin, 50 mg; D-calcium pantothenate, 25 mg; Cu (as copper sulfate), 150 mg; Fe (as ferrous sulfate), 100 mg; Mn (as manganese oxide), 40 mg; Zn (as zinc oxide), 100 mg; I (as potassium iodide), 0.5 mg; and Se (as sodium selenite), 0.3 mg.

^2)^Calculated value for DE, and analyzed values for other nutrients.

### Sample collection

When the feeding test ended, all pigs were anesthetized by intravenous injection of sodium pentobarbital solution. The gastrointestinal tract was removed, and the small intestine was separated from the large intestine and the mesentery, and rinsed thoroughly with ice-cold physiological saline solution. The middle segment of jejunum (2 cm) was collected and fixed in 4% formaldehyde for morphology analyses. Jejunal mucosa was also collected and immediately snap-frozen in liquid nitrogen and stored at -80°C for the analysis of gene expression. Then, the colon was quickly removed and the luminal contents were collected from a region 10 cm posterior to the ileocecal valve into sterile tubes and immediately stored at -80°C for the determination of intestinal microbiota composition and VFA concentrations [21].

### Growth performance

Body weights and feed intake were recorded at the beginning and end of the 28-day experiment period. Those data were used for calculating average daily gain (ADG), average daily feed intake (ADFI), and the feed: gain ratio (F/G) as previously described [22].

### Diarrhea frequency

The diarrhea frequency was conducted and determined as previously described [22]. Briefly, we monitored fecal consistence twice daily and scored it using a scale ranging from 0 to 3. 0, 1, 2, and 3 stand for normally shaped feces, shapeless (loose) feces, liquid (thick, soft) feces, liquid feces (thin, watery diarrhea), respectively. The piglet was regarded to have diarrhea when the scoring was bigger than 1.

### Intestinal morphology

The jejunal morphology of all the piglets were analyzed using hematoxylin-eosin staining as previously described [23]. Villus length and crypt depth were determined by Image-Pro Plus software (Media Cybernetics, Rockville, MD, USA), Version 6.0 on images at 200- or 400-fold magnification in ten randomly selected fields, respectively.

### Reverse transcription and real-time quantitative PCR

The quantitative RT-PCR analysis was conducted as previously described [24]. Briefly, total RNA was extracted from the jejunum using Trizol reagent (Invitrogen, Carlsbad, CA, USA). Primers for the selected genes were designed using the Oligo 6.0 software (**Table 2**). The house-keeping gene β-actin was used as internal control to normalize the expression of target genes. The relative quantification of gene amplification by RT-PCR was performed using the value of the threshold cycle (Ct). Relative expressions of target genes were determined by the 2^-△△Ct^ method.

**Table 2:** Primers used for quantitative reverse transcription-PCR.

Genes	Primers	Sequences (5’-3’)	Accession No.
ZO-1	Forward	TACCCTGCGGCTGGAAGA	XM_005659811.1
	Reverse	GGACGGGACCTGCTCATAACT	
Occludin	Forward	AGAGTCATAAGGTGGGGCAGT	NM_001163647.2
	Reverse	CGCCCGTCGTGTAGTCTGTC	
Claudin-3	Forward	CATCGGCAGCAGCATTATC	NM_001160075
	Reverse	ACACTTTGCACTGCATCTGG	
β-actin	Forward	GGATGCAGAAGGAGATCACG	XM_003124280.3
	Reverse	ATCTGCTGGAAGGTGGACAG	

### 16S rDNA gene sequencing with Illumina MiSeq sequencing

The 16S rDNA gene sequencing with Illumina MiSeq Sequencing was performed as previously described [25]. Briefly, the total bacterial DNA of colonic content was extracted according to the protocol of Qiagen QIAamp DNA Stool Mini Kit (Qiagen, Germany). 16S rDNA analysis genes were used to determine intestinal microbiota composition in each colonic sample. The V4 region of the 16S rRNA genes were selected since that at the 3% dissimilarity, the V4 region showed sufficient intergenomic variation and the least intragenomic heterogeneity in bacteria [26], and then targeted by purified microbial genomic DNA through the amplification of PCR with primers 515F (5’-GTGCCAGCMGCCGCGGTAA-3’) 806R (5’-GGACTACHVGGGTWTCTAAT-3’). Illumina MiSeq platform was used to analyze the data of sequencing and general data analyses. The platform and manufacturer's instructions were provided by commercial biology company (BGI Shenzhen Science and Technology Service Co., Ltd. Shenzhen, China).

Raw data were processed using QIIME (Version 1.80) and FLASH (Version 1.2.11) software packages. The UPARSE (Version 7.0.1090) was performed to cluster the OTU at 97% sequence similarity according to the combination of overlapped paired-end reads. Besides, chimeras were detected and dislodged using UCHIME (Version 4.2.40). Then, we employed the Ribosomal Database Project (RDP) Classifier (Version 2.2) to taxonomically classify the OTU representative sequences. The RDP Classifier was trained on the Greengenes database using 0.60 confidence values as cutoff besides. The complexity of species diversity is analyzed by the Observed-species, Chao1, ACE, Shannon and Simpson indices which are calculated by Mothur (Version 1.31.2, http://www.mothur.org/wiki/Calculators), and the corresponding rarefaction curve are drawn by software R (Version 3.1.1). The species diversity of phylum and genus levels in each sample were generalized in a histogram using the software R (Version 3.1.1).

### VFA analyses

VFA analyses were performed as previously described [25]. Briefly, after weighing, the colonic content (0.5~0.6 g) were placed in 10 mL centrifuge tubes. Then, 8 mL double-distilled H_2_O was added to 10 mL centrifuge tubes, mixed, homogenized, and centrifuged in sealed tubes at 15, 000 × g, 4°C for 15 min. As a result, the supernatant (0.9 mL) was transferred into a sealed 2 mL tube and mixed with 25% metaphosphoric acid solution (0.1 mL) by the volume proportion of 1:1, subsequently standing at 4°C for over 3 h. The supernatant fluid was then measured by gas chromatography using a UV-2450 spectrophotometer of 550 nm wave length (Shimadzu, Japan).

### Statistical analyses

Data obtained was analyzed by the One-way analysis of variance (ANOVA) using SAS 8.2 software (Cary, NC, USA) followed by a Duncan’s multiple comparison test. Results are presented as means with standard errors. Differences between significant means were considered as statistically different at *P* < 0.05 and a trend toward significant at *P* ≤ 0.10.

## Results

### Growth performance and diarrhea rate

**Fig 1:**
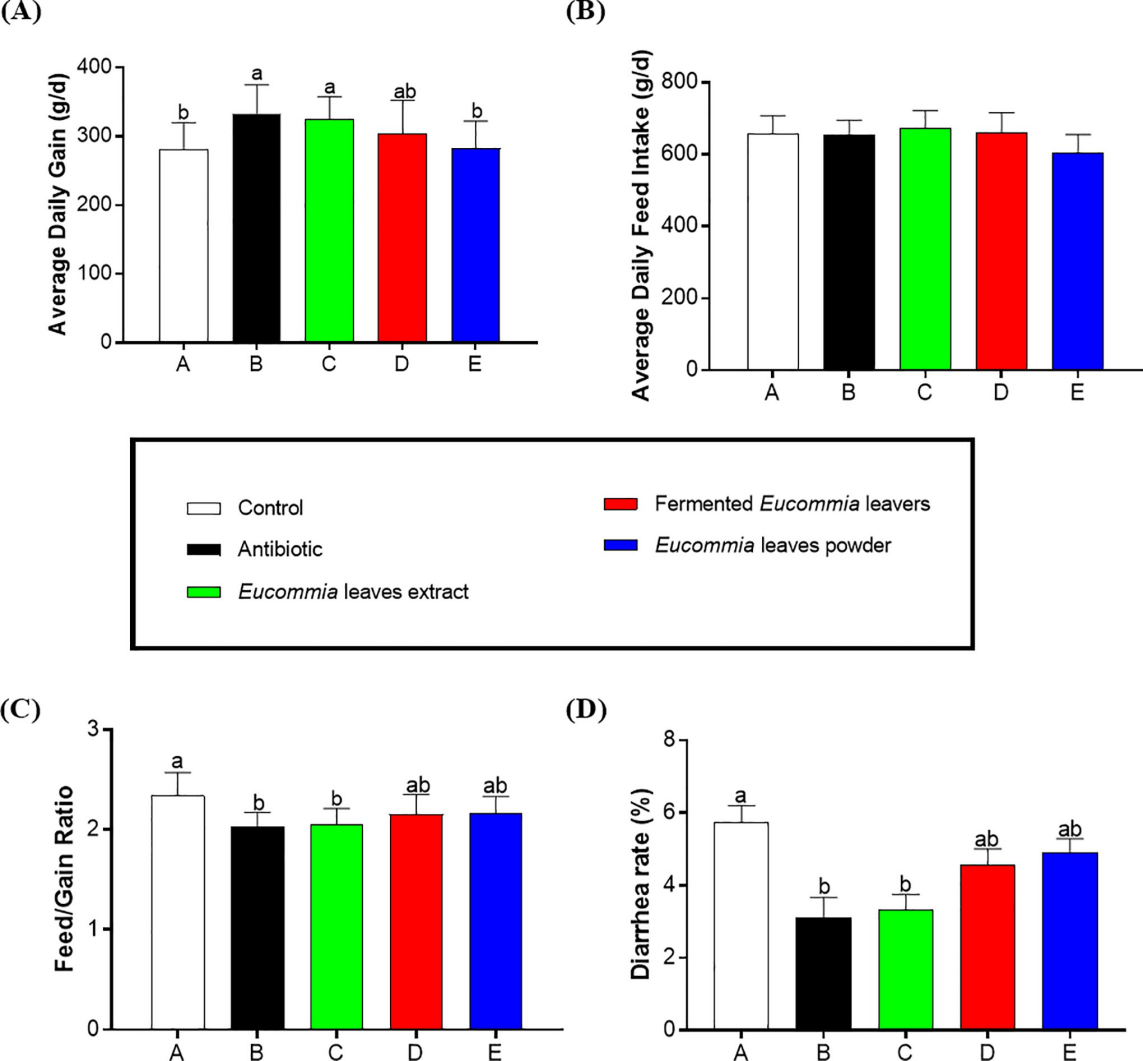
Growth performance. A: Average Daily Gain; B: Average Daily Feed Intake; C: Feed/Gain Ration; D: Diarrhea rate.

As revealed in **Fig 1**, compared to the control group, the antibiotic and EL extract groups showed a higher ADG and a lower feed: gain ratio as well as a lower diarrhea rate (*P* < 0.05), and there was no difference between the antibiotic and EL extract groups (*P* > 0.05). The fermented EL and EL powder groups did not change these parameters relative to the control group (*P* > 0.05). No significant difference in ADFI was observed among all groups (*P* > 0.05).

### Intestinal morphology in weanling piglets

**Fig 2:**
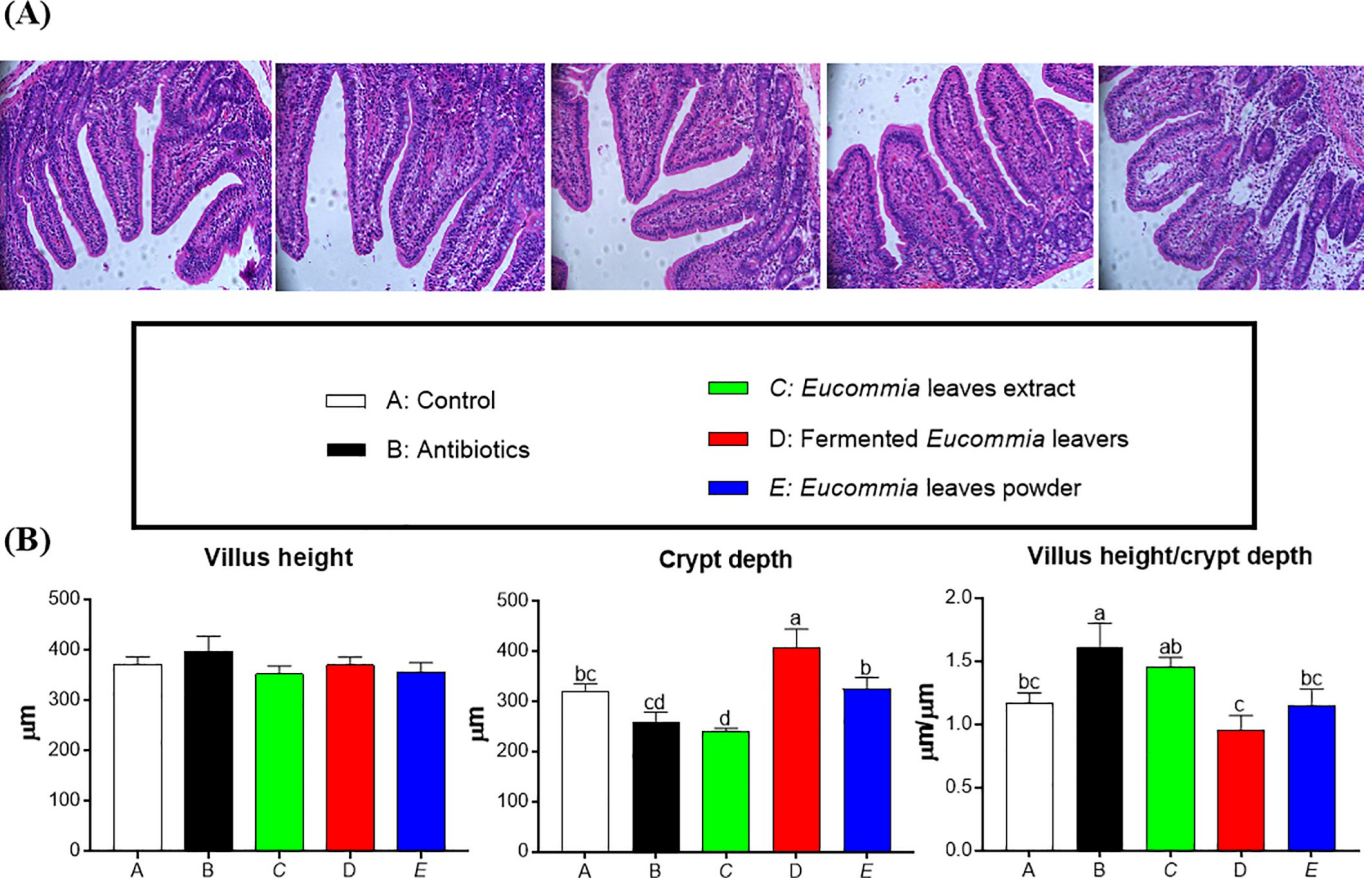
The jejunal morphology in weanling piglets. (A) Representative images of jejunal morphology. (B) Villus height, crypt depth, villus height/crypt depth in jejunum. Data are presented as mean ± SEM. ^a,b,c^ Mean values with different letters were considered to be significantly different (*P* < 0.05). The groups represented as follows: A: the control group; B: the antibiotic group; C: the *Eucommia ulmoides* leaves (EL) extract group; D: the fermented EL group; E: the EL powder group.

As shown in **Fig 2**, compared to the control group, the crypt depth was decreased and increased in the EL extract and fermented EL groups, respectively (*P* < 0.05). Compared to the antibiotic group, the fermented EL and EL powder groups exhibited a higher and a lower crypt depth, respectively (*P* < 0.05). No difference in crypt depth was observed between the antibiotic and EL extract groups (*P* > 0.05). The villus height was not influenced by dietary treatments (*P* > 0.05). The ratio of villus height to crypt depth in the EL extract group has similar value to the antibiotic group, and was higher than that in the fermented EL and EL powder groups (*P* < 0.05).

### The mRNA expression levels of claudin, occludin, and ZO-1 in the jejunal mucosa of piglets

**Fig 3:**
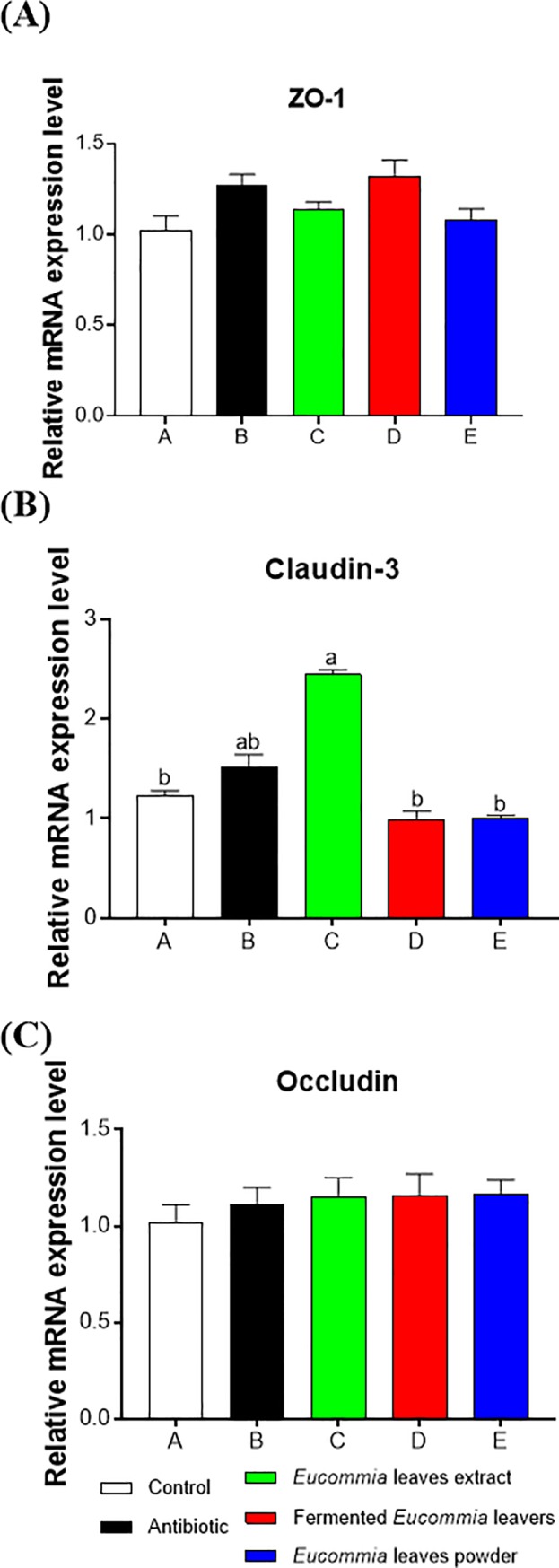
Relative mRNA levels of genes (ZO-1, occludin, claudin-3) in the jejunum of weanling piglets. The real-time PCR method was employed, and β-actin was used as an internal control. Values are means, with their standard errors represented by vertical bars (n = 8). ^a,b^ Mean values with different letters were considered to be significantly different (*P* < 0.05).

The relative mRNA expressions of tight junction proteins (ZO-1, claudin-3, and occludin) in the jejunum of weanling piglets are shown in **Fig 3**. Compared to the control and antibiotic groups, the EL extract group significantly increased the mRNA abundance of claudin-3 in the jejunum (*P* < 0.05), and the fermented EL and EL powder groups did not exert any effects (*P* > 0.05). The mRNA abundance of ZO-1 and occluding in the jejunum were not significantly different among the groups (*P* > 0.05).

### Colonic bacteria richness and diversity by alpha-diversity analysis

The OTUs and statistical estimates of species richness (Chao and ACE) and diversity (Shannon and Simpson) for each group at a genetic distance of 3% were revealed in **Table 3**, respectively. In the colonic contents, compared to the control and antibiotic groups, the EL extract group significantly increased the OTUs, Chao 1, and ACE indices (*P* < 0.05), and there was no difference among the other groups. Compared to the antibiotic group, the EL extract and fermented EL groups significantly enhanced the Shannon index (*P* < 0.05). The Shannon index was not significantly different between the control and the different forms of EL groups (EL extract, fermented EL, and EL powder groups), but tended to be higher in the EL extract group. The Simpson index was highest in the EL extract and fermented EL groups and lowest in the antibiotic group, with intermediate values in the other two groups (*P* = 0.07).

**Table 3:** Alpha diversity indices of colonic bacterial communities in weanling piglets.

Items	A	B	C	D	E	SEM	*P*-value
OTUs	1,871.33^b^	1,777.83^b^	2,337.50^a^	1,844.33^b^	1,595.33^b^	6.70	0.02
Chao 1	4,727.86^b^	4,561.73^b^	5,789.98^a^	4,318.89^b^	3,578.08^b^	10.94	<0.01
ACE	4,406.47^b^	4,359.37^b^	5,513.89^a^	4,023.85^b^	3,459.46^b^	10.69	0.01
Shannon	6.99^abc^	6.43^c^	7.50^a^	7.37^ab^	6.67^bc^	0.28	0.03
Simpson	0.97^ab^	0.95^b^	0.98^a^	0.98^a^	0.96^ab^	0.05	0.07

Notes: The OTUs were defined with 97% similarity. Means within a row with different superscripts are significantly different (*P*<0.05). A: the control group; B: the antibiotic group; C: the *Eucommia ulmoides* leaves (EL) extract group; D: the fermented EL group; E: the EL powder group.

### Colonic bacteria community composition by Illumina MiSeq sequencing analysis

**Fig 4:**
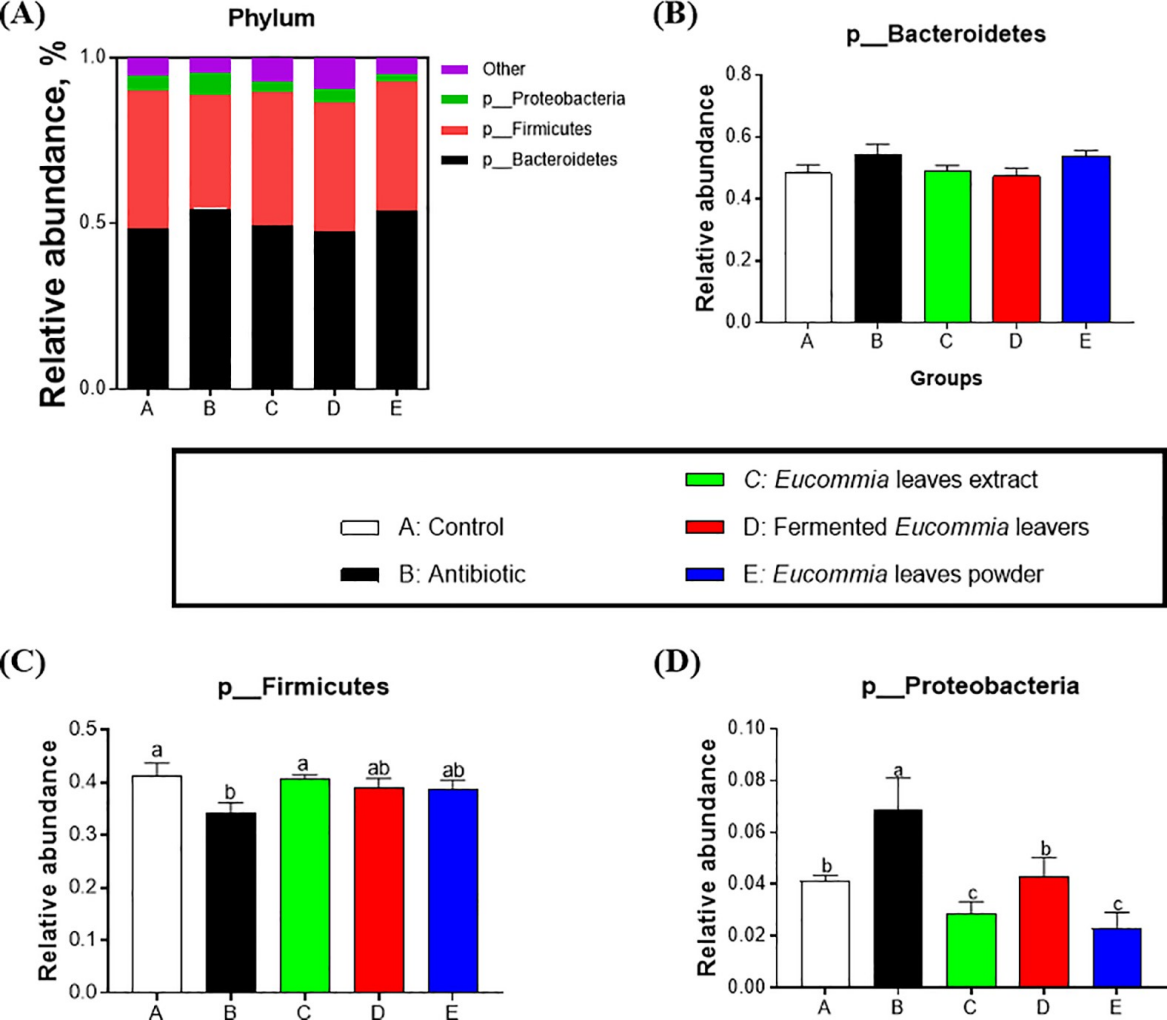
Relative abundance of colonic microbial communities at the phylum level in weanling piglets. The groups represented as follows: A: the control group; B: the antibiotic group; C: the *Eucommia ulmoides* leaves (EL) extract group; D: the fermented EL group; E: the EL powder group.

**Fig 5:**
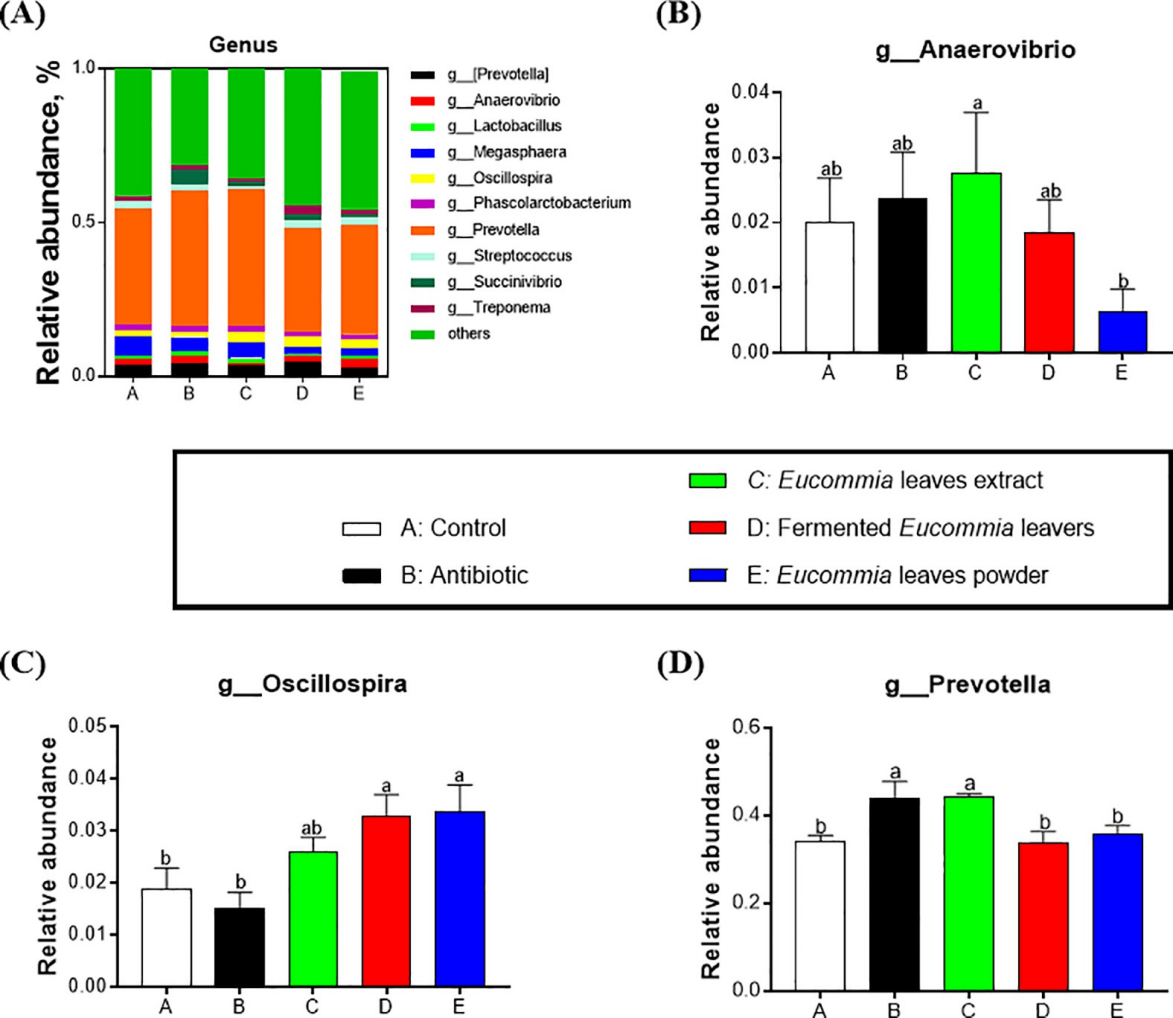
Relative abundance of colonic microbial communities at the genus level in weanling piglets. The groups represented as follows: A: the control group; B: the antibiotic group; C: the *Eucommia ulmoides* leaves (EL) extract group; D: the fermented EL group; E: the EL powder group.

The bacterial community composition in colonic content at the phylum and genus levels were shown in **Figs 4 and 5**. At the phylum level, twenty-one phyla were identified in total colonic samples and the abundance of three phyla among them was ≥ 0.5%, including bacteroidetes, firmicutes, and proteobacteria (**Fig 4**). In particular, bacteroidetes and firmicutes accounted for a relative abundance of 47.45–54.54% and 34.13–40.63%, respectively, followed by proteobacteria, at 2.28–6.85%. Compared with the antibiotic group, the EL extract group significantly decreased proteobacteria (*P* < 0.05). At the genus level, twenty-eight genera were detected in total colonic samples. Especially, there were ten genera with the abundance ≥ 0.5% (**Fig 5**). Among the genera, the genus *Prevotella* was the most abundant genera, accounting for 33.82–44.21%. Moreover, the *Prevotella* abundance was greatly elevated by the EL extract group relative to the control group (*P* < 0.05, **Fig 4D**).

### VFA concentrations

As revealed in **Table 4**, compared to the antibiotic group, the EL extract and fermented EL significantly increased the acetate concentration (*P* < 0.05). Compared to the control group, the EL extract exhibited an increased concentration of butyrate (*P* < 0.05). In addition, the EL extract increased the butyrate concentration by 26.47% (*P* > 0.05). Compared to the control and antibiotic groups, total VAF was significantly augmented in the EL extract group (*P* < 0.05).

**Table 4:** Concentrations of volatile fatty acids in the colonic content of weanling piglets.

Items	A	B	C	D	E	SEM	*P*-value
Acetate	22.52^ab^	21.36^b^	26.07^a^	25.61^a^	23.41^ab^	2.35	0.02
Propionate	8.54	9.45	10.06	10.15	10.84	0.16	1.23
Isobutyrate	0.32	0.47	0.36	0.35	0.42	0.06	0.36
Butyrate	5.60^b^	6.31^ab^	7.98^a^	6.06^ab^	6.35^ab^	0.24	0.03
Isovalerate	0.40	0.51	0.42	0.43	0.42	0.06	0.35
Valerate	0.62	0.58	0.66	0.64	0.62	0.09	0.78
Total VFA	38.00^b^	38.68^b^	45.55^a^	43.24^ab^	42.06^ab^	3.12	0.04

Means within a row with different superscripts are significantly different (*P*<0.05). A: the control group; B: the antibiotic group; C: the *Eucommia ulmoides* leaves (EL) extract group; D: the fermented EL group; E: the EL powder group.

## Discussion

The current study for the first time indicates that after a 4-week supplementation, the EL extract, but not the fermented EL or EL powder, markedly improved the overall growth performance and decreased the diarrhea rate in weanling piglets. These findings are in accordance with studies using other animal models such as fish [27] or poultry [28], which reports that the EL extract can promote the growth rate/performance. Although the reason why the EL extract is superior to the other two forms of EL (fermented EL and EL powder) is not clear, it is possible that bioactive substances in the EL extract are higher than those in the fermented EL and in the EL powder (such as total flavonoids in the EL extract, fermented EL, and EL powder are > 8.00%, 4.30%, and 7.01%, respectively). Notably, we further found that the beneficial effects of the EL extract reach the levels comparable with antibiotics. Taken together, our data suggest that the EL extract has the potential to replace antibiotics in the prevention of diarrhea in weanling piglets and to improve their growth performance.

The small intestine is the primary organ for digesting and absorbing luminal nutrients, hence their integrated mucosal structure is essential for nutrient absorption and optimal growth [29]. Notably, weaning stress will lead to alteration of the intestinal mucosal structure and function [30]. Data from this study showed that the EL extract decreased the crypt depth and increased the ratio of villus height to crypt depth to the levels similar to the antibiotics, suggesting an elevation in the absorption area of the intestinal mucosa. In addition to improving small intestinal morphology, the EL extract supplementation may reduce intestinal permeability, as manifested by increases in mRNA expression of the tight junction proteins. It is reported that weanling stress triggers sustained impairment in the intestinal barrier characterized by downregulated expression of tight junction protein [31, 32]. The mRNA expression of claudin-3 was elevated in the jejunum of the EL extract-treated piglets in this study, suggesting its roles in the intestinal epithelial barrier function. Consistent with the data of growth performance and diarrhea rate, we observed that neither the fermented EL nor the EL powder improved the jejunal morphology and permeability. Therefore, we speculate that improved growth performance of piglets fed the EL extract-supplemented diets may be associated with improved small intestinal structure and function.

Next, we further explored the effects of dietary supplementation with different forms of EL on the cecal microbial community composition and diversity in weanling piglets. Intestinal microbiota influences feed conversion, nutrient absorption, growth, and epithelial development immunity along with the intrusion of pathogenic microorganisms [33]. In this study, dietary supplementation with the EL extract was superior to the fermented EL and the EL powder in effectively elevating colonic microbial community richness (Chao 1, ACE) and diversity (Shannon, Simpson). Intestinal microbiome exerts significant roles in host nutrition and health, feed intake and efficiency as well as weight gain by interacting with nutrient utilization and the development of gut system of the host [1, 34, 35]. This study showed that dietary supplementation with the EL extract decreased the abundance of the phylum *Proteobacteria* and increased the abundance of *Prevotella* genus, whereas the antibiotics treatment increased the *Proteobacteria* abundance. As a front-line responder, *Proteobacteria* responds sensitively to environmental factors, such as diet. A bloom of the intestinal *Proteobacteria* abundance indicates dysbiosis or an imbalanced gut microbiota [36]. Moreover, the antibiotics treatment has been reported to lead to dysbiosis indicated by reducing intestinal microbiota diversity and taxonomic richness [37]. These results suggest that the EL extract is superior to antibiotics in enhancing beneficial bacterial species and improve the compositions of the intestinal microbiota in weanling piglets. Reduced population of pathogenic bacteria may enhance availability of nutrients, ameliorate sub-clinical infections, and decrease generation of growth-depressing toxins or metabolites by intestinal microbiota [1]. Recent studies indicated that the growth-promoting effects of antibiotic was associated with the reduced activity of bile salt hydrolase, an intestinal bacteria-produced enzyme that plays detrimental effects on host fat digestion and utilization [38]. Growth enhancement through the utilization of the EL extract is probably due to the synergistic effects among complex active molecules existing in the EL extract. As expected, the EL extract has a very complicated blend of bioactive components, including polysaccharides (> 20.00%), total flavonoids (> 8.00%), and chlorogenic acid (> 5.00%). Polysaccharides extracted from EL is mainly composed of glucose, fructose, mannose, fucose, galactose, and arabinose, and is a strong immunostimulant that can strongly enhances immune responses [39]. Polysaccharides obtained from the herb *Astragalus membranacea* Radix and two mushrooms *Lentinus edodes* and *Tremella fuciformis*, appear to be potential alternatives for antimicrobial growth and health promoters. These products might exert a critical role in strengthening the immune system by improving the physical conditions of gut ecosystem and augmenting functions of the defensive system of animals [40]. Our data suggested that these bioactive components existing in the EL extract might influence the growth of weanling piglets through improving gut microbial composition and diversity and reducing the pathogenic bacteria *Proteobacteria*.

It has been reported that intestinal microbiota has the ability to rapidly shape intestinal fermentation of nutrients such as proteins and carbohydrates to produce VFA, which is closely related to intestinal metabolisms [41, 42]. In addition, *Prevotella* exerts beneficial effects on fermentation and hydrolysis of luminal carbohydrate and protein, generating propionate and acetate [43]. Results from this study showed that colonic concentrations of acetate, butyrate, and total VFA were greatly elevated in response to dietary supplementation with the EL extract. Lower acetate levels might give rise to the inhibition of lipogenesis [44]. Butyrate is regarded to be an important source of energy for intestinal epithelial cells and exerts beneficial effects on anti-carcinogenesis, anti-inflammation, decreasing oxidative stress, and improving intestinal barrier [45]. The VFA, the primary products of intestinal microbiota, is produced from the fermentation of nutrients such as peptides, proteins, and carbohydrates [46], and modulates intestinal physiology and metabolism [47]. Generally, the enzyme that degrades nutrients such as carbohydrates is secreted by intestinal microbes. Short-chain fatty acids are mainly produced by beneficial bacteria such as lactic acid bacteria and bifidobacteria in the colon from non-digestible sugars (non-digestible oligosaccharides, non-starch polysaccharides and resistant starches). And the final fermentation products are organic fatty acids that are more easily absorbed. such as acetic acid, propionic acid and butyric acid. Meanwhile, volatile fatty acids are important organic acid anions in the colon, which have the functions of maintaining intestinal morphology, maintaining the balance between body fluids and electrolytes, and regulating intestinal flora. The types and quantities of volatile fatty acids are often affected by factors such as the fermentation substrate, type, degradation rate, degree of degradation, intestinal microorganisms and host physiological status. Therefore, the level of short-chain fatty acids in the intestine can be used to evaluate the changes of anaerobic bacteria, and the content of volatile fatty acids can also reflect the activity of intestinal microorganisms [46–47]. Overall, we suggested that dietary supplementation with the EL extract promoted bacterial fermentation to modulate intestinal microbiota and VFA levels in the intestinal tract.

## Conclusion

In summary, dietary supplementation with the EL extract, but not the fermented EL and the EL powder, could improve growth performance, jejunul morphology as well as function, and changed colonic microbial composition and diversity of weanling piglets, with a concurrent reduction of diarrhea rate (**Fig 5**). Moreover, the effects of the EL extract were similar to those of antibiotics. Although the precise mechanisms are not clear, our results demonstrate the feasibility of using the EL extract as natural green dietary additives for weanling piglets to be an alternative of feed antibiotics, thus conferring protection against weanling stress injury. Further investigation is warranted to prove whether the EL extract treatment under conditions of weaning stress is beneficial in the whole-body context and in a long-term use.
